# Selection of Forage Resources by Juvenile Goats in a Cafeteria Trial: Effect of Browsing Experience, Nutrient and Secondary Compound Content

**DOI:** 10.3390/ani12101317

**Published:** 2022-05-21

**Authors:** Gabriel Andrés Ortíz-Domínguez, Cindy Goretti Marin-Tun, Rafael Arturo Torres-Fajardo, Pedro Geraldo González-Pech, Concepción Manuela Capetillo-Leal, Juan Felipe de Jesús Torres-Acosta, Javier Ventura-Cordero, Carlos Alfredo Sandoval-Castro

**Affiliations:** 1 Facultad de Medicina Veterinaria y Zootecnia, Universidad Autónoma de Yucatán, Carretera Merida-Xmatkuil km 15.5, Merida 97315, Mexico; gabriel.ortiz.mvz@gmail.com (G.A.O.-D.); cindymarintun@gmail.com (C.G.M.-T.); rafael-arturo-torres@outlook.es (R.A.T.-F.); petergonzyuc@gmail.com (P.G.G.-P.); concepcion.capetillo@correo.uady.mx (C.M.C.-L.); tacosta@correo.uady.mx (J.F.d.J.T.-A.); 2 School of Biological Sciences, Queen’s University Belfast, Chlorine Gardens, Belfast BT9 5BL, UK; j.ventura-cordero@qub.ac.uk

**Keywords:** intake, small ruminants, native foliage, forage availability

## Abstract

**Simple Summary:**

Grazing/browsing goats ingest a diverse diet selected from the forage resources available in the range/paddock intending to meet their nutritional requirements. Intake and selection of any given forage can be modified by several factors, such as its nutrient content, including secondary compounds, as well as its biomass availability in the environment. The animal’s previous browsing experience can also be an important factor driving intake and selection. Therefore, the present study evaluated all these factors as well as their interaction. The results showed that the goats’ browsing experience guided the selection and intake towards those forage resources of better nutritional quality when there was no restriction in forage supply, as is the case for cafeteria trials. Goats with browsing experience showed their ability to limit secondary compounds and optimize their selection and intake for plants with better digestibility. This cafeteria protocol made it possible to identify the selection and consumption pattern of plant species with limited availability in the natural vegetation. This methodology could help identifying the forage resources that may be useful for small ruminant feeding, versus those resources that are not consumed by goats despite having a suitable chemical composition and in vitro digestibility or a high abundance.

**Abstract:**

We evaluated the effect of browsing experience, nutritional quality and secondary compounds of forage resources, and the interaction between these factors on the selection and intake of goats in a cafeteria trial. Twelve juvenile Criollo goats from 7 to 9 months of age, weighing 22 ± 3 kg, were divided into two groups: (a) browser goats group (*n* = 6, BG), and (b) naïve goats group (*n* = 6, NG), formed according to their previous browsing experience (with and without, respectively). Animals were housed in individual pens. The cafeteria experiment lasted 21 days considering pen adaptation, foliage adaptation, and measurements, which included the selection index (SI) of experimental forage resources (Chesson’s alpha) and their dry matter intake (DMI/Kg^0.75^), using a multiple Latin square design. Furthermore, correlation and regression analyses were used to assess the relationship between the aforementioned factors. The NG did not show any selection pattern, while the BG selected *Piscidia piscipula* and *Senegalia gaumeri* (*p* = 0.0002). The BG consumed smaller amounts of secondary compounds compared to NG (*p* = 0.0001). In the BG, the flavonoids affected negatively their selection (R^2^ = 97.51, *p* = 0.0001), while the DMI was affected by in vitro DM digestibility and flavonoids (R^2^ = 99.85; *p* = 0.0001). For the NG, the crude protein and organic matter contents were associated with DMI, but none had a significant relationship with SI. The BG selected and consumed forages with suitable nutritional quality avoiding those with high content of secondary compounds such as flavonoids. Conversely, NG did not show a clear pattern for their selection or intake.

## 1. Introduction

Dietary selection and intake of a diversified diet generally allow browsing herbivores to cover their nutritional requirements [1], although it may not be the case under specific circumstances, as shown recently for goats browsing the Mediterranean forest [2]. However, several factors modify the selection and intake of plant resources consumed by goats. Under some conditions, the higher the abundance of a given resource in the field, the more likely it is that goats will select and consume that resource [3,4]. The latter agrees with the optimal foraging theory which indicates that a greater presence of any resource in the patch allows a lower expenditure of energy used to search for food, and goats will remain for longer periods of time in that same patch [5]. On the other hand, when there is low biomass availability, goats may extend grazing time (increasing their search for food) to compensate for the low intake rate [6]. Thus, goats’ behavior can be an example of the model for a “patchy habitat” proposed by Charnov [7]. Feeding behavior studies in the heterogeneous vegetation of the tropical deciduous forest have shown that goats can consume up to 51 plant species during the rainy season [8] and up to 33 species during the dry season [9]. Among these plants, some species have been identified as relevant forages for browsing goats due to their high relative importance value (biomass availability) in the tropical deciduous forest ecosystem [10]. Meanwhile, some plant species seem to be selected, but animals only achieve a low intake. It is unknown whether the observed intake and selection by goats was constrained by their biomass availability or if other factors are involved in modifying selection and intake.

Intake can be related to several aspects of the plants, i.e., their chemical composition and content of secondary compounds (SC) [11,12], which may encourage animals to seek plant materials that allow a higher nutrient intake while minimizing the consumption of SC as the latter could reduce their intake and productivity (i.e., flavonoids [13,14], alkaloids and saponins [15,16,17]).

The biomass density (seasonal spatial arrangement) and plant morphology (shape) of leaves can induce goats to favor the selection of those plants that allow faster intake rates as it is more efficient to harvest them [4,18]. Plants that allow lower intake rates, either due to increased bite size (i.e., large leaves), low density (voluminous shape) or the presence of thorns, are generally less selected by small ruminants [12,19,20].

Browsing experience is an additional factor that could influence the use of forage resources. For example, kids’ learning explained better the use of tannin-rich plants than the genetic responses [21]. The acquisition of browsing experience at an early age triggers a series of changes (morphological, physiological, and neurological), providing a better adaptation to the environment and influencing the animals’ behavior [22]. Thus, naïve or inexperienced animals, accustomed to use a limited number of feeds within a controlled environment, are less likely to explore new plants even to meet their nutritional requirements. On the other hand, experienced animals, which have learned through consequences, can learn about new feeds and choose beneficial combinations of feeds, extending their behavior towards their offspring [1,23].

Similarly, juvenile goats fed with low-quality forages before weaning can use their experience to improve the selection of specific resources [24]. In addition, early exposure to SC can increase the ability of goats to face their challenge, representing a novel alternative to prevent diseases in ruminants [22,23]. Thus, Provenza and Malechek [25], studying the resource selection of adult and juvenile goats fed on rangeland, concluded that the diets of these groups were similar in terms of the leaf:stem ratio and crude protein (CP) content, but juvenile goats spent more time searching for food in areas with low diversity. Recently, González-Pech et al. [26] reported that experienced adult goats and inexperienced kids have similar dry matter intake (DMI per kg BW), and their diets contained similar amounts of grass and shrubs when browsing the tropical forest. However, kids consumed more plants from the lower strata, displayed smaller bites, and consumed fewer plant species than their adult counterparts. Thus, the browsing behavior of goats can be modified not only by plant factors or biomass availability but also browsing experience seems to be relevant. The latter can be learned from their adult counterparts [25,26] and seems to be an important aspect to be considered.

However, to the best of our knowledge, the effect of browsing experience upon preference has not been studied under controlled conditions in sheep or goats. In addition, reliable estimations of intake or preference are difficult to obtain for plant species with low biomass availability in the field (low relative importance value or RIV), as the possibility of confounding effects between RIV and intake or preference cannot be ruled out. Furthermore, there are no studies focusing on contrasting and validating intake and preference results obtained under field grazing/browsing vs. cafeteria trials results.

The cafeteria methodology is a valuable tool that can allow studying, under controlled conditions, those factors influencing plant selection/preference and intake, thus, contributing to the understanding of ruminants’ feeding behavior [27,28].

Therefore, the objective of this experiment was to evaluate in a cafeteria trial the effect of browsing experience, nutritional quality, and secondary compound contents of six forage resources (selected according to their contrasting RIV, intake, and preference) on the selection and intake of juvenile goats.

## 2. Materials and Methods

### 2.1. Study Site

The study was conducted at the Faculty of Veterinary Medicine and Animal Science–Universidad Autónoma de Yucatán, México (FMVZ-UADY) (20°52′7.14″ N and 89°37′24.04″ W). The collection of plant species was carried out in the surrounding tropical forest. The prevailing climate in the zone is sub-humid, warm with rains in summer (AW_0_), average annual temperature of 26 °C, with a thermal oscillation of 5 to 7 °C [29].

### 2.2. Forage Resources

Six forage resources were included in the study based on previous reports of goats’ DMI and preference in the tropical deciduous forest [8,9]. Their biomass availability in the field based on its RIV was also considered. The latter measures the impact of plants within an ecosystem (dominance, density, and relative frequency in the field). According to Dzib-Castillo et al. [10], forage species with a RIV > 12 have the most significant impact, and those species < 12 are those with the lowest impact within the ecosystem.

Plants were selected to represent contrasting examples of RIV, intake, and preference [30,31]. Thus, the selected forage resources were classified into:

1. RIV < 12 and low intake by goats (DMI < 1% live weight (LW)): *Caesalpinia gaumeri* Greenm., *Cordia alliodora* Oken, *Neomillspaughia emarginata* H. Gross SF Blake, *Senegalia gaumeri* SF Blake. There are no previous selection reports due to their limited availability and low DMI;
2. RIV > 12 and high intake by goats (DMI > 1% LW): *Gymnopodium floribundum* Rolfe with a negative/low selection index (SI) under browsing conditions and *Piscidia piscipula* L. Sarg. with a positive/high SI under browsing conditions.

For a representative sample, forage resources were harvested twice a week (8:00–11:00 h) manually from January to September 2018. The leaves and small diameter stems were used, eliminating the thick branches and flowers (if any). The forages were pre-dried in a barn and then were dried in a forced-air oven (38 °C) until a constant weight was achieved. Dried samples of plant materials were stored at room temperature in sealed plastic containers until their use.

### 2.3. Experimental Goats and Their Management

All experimental procedures complied with ethical standards based on the guidelines published by Sherwin et al. [32]. In addition, the experimental protocol was examined, validated, and approved by the Bioethics Committee of the FMVZ-UADY (No. CB-CCBA-M-2019-003).

Twelve female juvenile goats (local Criollo goats) from 7 to 9 months of age (22 ± 3 kg of LW) were divided into two groups (*n* = 6): (a) Browser goats group (BG) that included juvenile goats with browsing experience; and (b) Naïve goats group (NG) that included naïve juvenile goats without browsing experience. The BG had more than five months of browsing experience in the tropical deciduous forest. Meanwhile, in the NG group, the juvenile goats were purposely maintained without any previous contact with the tropical deciduous forest and had not consumed/browsed local plant species as they remained indoors, consuming a complete diet manufactured with grains and chopped grass (see below). To prevent experienced goats from having contact with their naïve counterparts before, during, or after the trial, they were placed in individual concrete floor pens (3 × 3 m). This was intended to avoid any change in feeding behavior that could be attributable to social influence during the experimental period.

Goats were fed with a grain-based balanced feed prior to offering the forages under evaluation. Finally, *Pennisetum purpureum* Schumach grass was offered later in the day to cover maintenance requirements for a LW gain of at least 40 g/d. Thus, the diet was designed to represent a DMI of 3.5% LW, avoiding bias attributed to animal appetite (empty rumen effect) but allowing goats to meet their low-activity maintenance requirements [33]. Water was offered *ad libitum*. The goats were dewormed before the start of the study using levamisole (12 mg/kg LW) and ricobendazole (10 mg/kg LW) to keep them free of gastrointestinal nematodes during the study.

Finally, all animals were weighed at the beginning and end of the cafeteria study periods to calculate the average daily weight gain for each experimental group.

### 2.4. Experimental Periods

#### 2.4.1. Pens Adaptation Period

This period lasted ten days. Animals were placed in individual concrete-floor pens. The feeding management included grain-based balanced feed to cover 1.0% of their LW in DM (250 g) and *P. purpureum* grass to cover 2.5% of their LW as DM (625 g/animal); these foods were offered in individual plastic containers for each animal with *ad libitum* provision of water.

#### 2.4.2. Adaptation to Forage Resources Period

This period lasted five days, during which the balanced feed was offered to cover 1.0% of its LW in DM (250 g) at 10:00 am. During this adaptation period, each of the six forage resources was offered to individual goats at 100 g DM, which is an amount higher than values previously reported for cafeteria trials with goats [34]. The forage of each plant species was offered in a plastic container that was placed on one side of the pen by the side of the other five containers with other plant species. Later (4:30 pm), *P. purpureum* grass was offered to cover 2.5% of its LW as DM (675 g). Refusals were weighed daily, and data were recorded for each individual plant material and feed.

#### 2.4.3. Cafeteria Measurement Period

The cafeteria trial period lasted six days. This methodology allowed to evaluate goat selection and intake as feeds were supplied *ad libitum* and with the possibility to freely decide which forage they wanted to ingest. At the beginning of the trial, 200 g DM for each forage resource, namely *C. alliodora, C. gaumeri, N. emarginata, S. gaumeri, G. floribundum*, and *P. piscipula* were placed in their respective plastic container and were also placed in one side of the pen. The selection and consumption observation protocols were carried out for 30 min on all the goats. This period of observation was based on previous studies that showed that the intake pattern observed along the first hour is maintained for up to 4 h of observation [11,12]. The goats were restrained inside their individual pen for 15 min before starting the period of intake and selection measurements to allow that all forage resources were placed inside the pens. The latter allowed to begin the cafeteria trial with all forages simultaneously available. The position of containers with each forage species was changed every day to avoid bias due to animals getting used to obtaining any particular forage from a specific pen position.

During the measurements period, six trained observers (one person observed two goats) monitored the intake of the different forage resources. An animal:observer habituation protocol was used [35] during a period of 13 days before the study, as recommended to adapt the goats to the handling of the observers. The success of the habituation protocol was confirmed when the goats allowed their observation at a distance of <1 m without interference from their feeding behavior inside the pen. These observers helped maintaining the *ad libitum* availability of the foliage in the different containers used for the plant species. They refilled (50 g DM per refill) containers before the edible material was depleted. At the end of the observation time, the goats were restrained again to one side of the pen to avoid further intake and to facilitate the collection of the remaining plant material. The remaining material was kept in the plastic containers and was weighed daily once the collection of all the containers was completed. To cover the nutritional requirements of experimental goats, concentrate feed and chopped *P. purpureum* grass were offered in the quantities and schedules employed during the adaptation period. The grass refusals were also weighed daily. Animals never refused any concentrate feed.

### 2.5. Voluntary Intake

The DMI per kg of metabolic LW (DMI/kg^0.75^) of the balanced feed, forage resources, and the *P. purpureum* grass was calculated as the difference between the quantities offered and refused (leftovers). Likewise, the intake of macronutrients and SC of each forage resource and the whole diet were estimated daily per animal for each group of goats.

### 2.6. Selection Index

The Chesson α index was employed as the selection index (SI), which takes into account that foliage availability varies over time. The resource availability could be considered constant as the food containers were refilled as the plant materials were consumed. This was performed by re-filling the forage species when necessary [36].

The Chesson α index was calculated as:

*αi* = (*oi*/*πi*)/∑ (*oi*/*πi*) (1)

where *α* was the Chesson SI for each forage resource *i*; *o* was the proportion of sample of units used in the diet (consumed) for each resource *i*; *π* was the proportion of sample of available units in each forage resource *i*.

The SI values vary between 0 and 1, measuring the strength of the forage selection per goat, the sum of all the α for all the plant species considered equal to the unit [36]. For this study, the non-selectivity point was *αi* = 0.16. The value *αi* = 0.16 arose from the number of plants (m) used in the preference study. It was calculated according to the formula (*αi* = 1/m), which indicated that the number of resources tested in this study was 1/6 = 0.16. Higher indexes (>*αi*) indicated a selection of forage resources, and lower indexes (<*αi*) indicated rejection.

### 2.7. Laboratory Analysis

The chemical composition of each forage species: CP, organic matter (OM), ether extract (EE), neutral detergent fiber (NDF), acid detergent fiber (ADF), lignin, in vitro dry matter (IVDMD), organic matter (IVOMD) digestibility, total phenols (TP), total tannins (TT), and condensed tannins (CT) were determined in the Animal Nutrition Laboratory at the FMVZ-UADY and previously reported by Ortíz-Domínguez et al. [37]. In addition, a phytochemical screening of the forage resources was performed to assess the presence of other SC: (i) alkaloids with Warner test, (ii) flavonoids with Alkaline test, (iii) monoterpenes with Lieberman-Buchard test, and (iv) saponins with foam test [38]. Additionally, SC were quantitatively measured: alkaloids [39,40], flavonoids [41], and saponins [40].

### 2.8. Foliage Density

The foliage density (FD) of each of the dry forage resources was estimated to identify whether the structure/morphology of the plant leaves of each plant species affected selection and intake. The FD was calculated using the following equation:

FD = *V/W* (2)

Where: *V* = volume of plastic container (in L) and *W* = amount occupied by each foliage resource (in g).

The volume of a plastic container was 13.5 L, and the forage weight was estimated by filling up to the edge of the container without pressing the fodder inside the container. The density was measured five times for each forage resource evaluated [20].

### 2.9. Statistical Analysis

The SI and DMI (g/kg^0.75^) of the forage resources tested were subjected to the normality tests of Shapiro–Wilk and respective tests of homogeneity of variance (Levene test).

A multiple Latin squares design 6 × 6 was used, where the column represented the day of the experiment, the row represented the position of each container (one species of foliage) inside the pen, the treatments were the 6 forage resources, and the animal represented the square. The browsing experience (BG and NG) was included in the model as a blocking factor.

The SI and the DMI did not present a normal distribution (*p* = 0.001). They were normalized when transformed to the natural logarithm (n + 1) for analysis. Subsequently, they were re-transformed to non-logarithmic values to present the results. The GLIMMIX PROC of the SAS program [42] was used for the SI and the DMI data. In the models, the distribution of the appropriate response variable (beta distribution) and the link function (logit) was specified, and a containment approximation was used for the degrees of freedom of the denominator.

The model was:

*yij*(*k*)*m* = *µ* + *SQm* + *ROW*(*SQ*)*im* + *COL*(*SQ*)*jm*+ *τ*(*k*) + *εij*(*k*)*m* (3)

where:

*yij*(*k*)*m* = SI or DMI of the goats

*µ* = the overall mean

*SQm* = the effect of experience (NG and BG) used as a block factor *m*

*ROW*(*SQ*)*im* = the effect of position of each plant container *i* within experience (NG and BG) *m*

*COL*(*SQ*)*jm* = the effect of day of experiment *j* within experience (NG and BG) *m*

*τ*(*k*) = the effect of each forage resource *k*

*εij*(*k*)*m* = random error with mean 0 and variance σ^2^

The intake data for CP, ADF, lignin, ME, OM (whole diet), and CT (whole diet and each forage species) were transformed to the natural logarithm (n + 1), and subsequently, they were back-transformed to non-logarithmic values to present the results. Analysis of variance with a completely randomized design was used to evaluate: (i) total intake (nutrients and SC) of the whole diet (concentrate feed + grass + the six forage resources) and (ii) intake (nutrients and SC) from forage resources only. Means for each of the evaluated nutrients and FD are presented for each experimental group (BG and NG). The *p* < 0.05 was considered statistically significant in all cases, and the Tukey test was used to compare means.

The association between the SI and DMI with the forage resource characteristics was determined separately for BG and NG using Pearson correlation analysis. The analyses included factors of the nutrient content (CP, EE, OM, NDF, ADF, Lig, and ME), SC (alkaloids, flavonoids, saponins, TT, CT, and TP), the IVDMD, IVOMD, and FD of each forage resource.

Finally, a STEPWISE proc was used to evaluate relationships (subset of best predictors). The respective ANOVA, correlation, and regression analyses were performed using the statistical software Minitab [43].

## 3. Results

During the whole experiment, goats did not present signs of toxicity from the forage ingested and had an average LW gain of 90 g/d. Therefore, they remained clinically healthy during the trial.

### 3.1. Nutritional Quality and Phytochemical Screening

Some data on the chemical composition and in vitro digestibility of the six forage resources were previously reported by Ortíz-Domínguez et al. [37]. In summary, CP content was above 100 g/Kg DM for the six species, with *S. gaumeri* as the forage with more CP. The *N. emarginata* and *G. floribundum* foliages had the highest fiber contents (NDF, ADF, and lignin), and the *C. alliodora* forage with the highest ME content. In addition, leaves from *C. alliodora* also exhibited the best IVDMD and IVOMD, contrasting with *N. emarginata,* which had the lowest values (Table 1). In all the forage resources evaluated, the presence of SC was detected, either by qualitative or quantitative tests. Alkaloids and flavonoids were present with a higher content in the forage of *C. gaumeri*, while *P. piscipula* had the lowest content. Regarding the content of saponins, the forage of *G. floribundum* showed a high quantity, and *C. gaumeri* exhibited the lowest content. The full chemical composition, including SC, IVDMD, IVOMD, and ME, is presented in Table 1.

The SC qualitative analysis found alkaloids only in the leaves of *G. floribundum* (+) and *N. emarginata* (+), while the other four species were negative (−). Flavonoids were detected in the leaves of *C. gaumeri* and *C. alliodora* (+++) > *G. floribundum* and *N. emarginata* (++) > *P. piscipula* (+) and *S. gaumeri* (−). Finally, saponins were detected in the following order: *N. emarginata* and *S. gaumeri* (>14 mm) > *C. alliodora* and *P. piscipula* (10–14 mm) > *C. gaumeri* and *G. floribundum* (<10 mm) by the foam test.

The density values of all the forages resources studied were different (*p* = 0.0001). The general pattern was *S. gaumeri* > *C. gaumeri* > *C. alliodora* > *G. floribundum* > *P. piscipula* > *N. emarginata* (0.059, 0.054, 0.045, 0.030, 0.027, and 0.015, respectively).

### 3.2. Selection Index in Juvenile Goats

An experience × foliage interaction was observed (*p* = 0.0001), where the forage of *P. piscipula* and *S. gaumeri* were selected by the BG, while the NG selection was directed toward *N. emarginata* and *C. gaumeri* (*p* = 0.0081 and = 0.024, respectively, Table 2). Both groups of goats selected *S. gaumeri* (*p* = 0.0002) and rejected *C. alliodora* (*p* = 0.678), *G. floribundum* (*p* = 0.168) with the same intensity (Table 2).

The NG did not show significant differences in the selection of the different forage resources evaluated (*p* > 0.05). Meanwhile, the BG showed differences for *P. piscipula* and *S. gaumeri* (both selected) in comparison to the other four forage species (*p* = 0.001, Table 2).

### 3.3. Feed Intake in Juvenile Goats

The BG consumed more of *P. piscipula* foliage, while *C. gaumeri* foliage was more consumed by the NG (experience × foliage interaction *p* = 0.0003, Table 3). In addition, within the NG group DMI of forage resources was similar (*p* > 0.05), while in the BG, the DMI of *S. gaumeri* and *P. piscipula* was higher (*p* = 0.001, Table 3).

The total intake (either dry matter or analyzed chemical fractions) of the whole diet (balanced feed, grass, and forages), as well as the total intake from the six forage resources, is shown in Table 4. Both groups of goats consume similar amounts of DM, CP, EE, OM, ADF, NDF, lignin, and ME (*p* > 0.05). However, the BG consumed less TP, TT, and CT than the NG (*p* < 0.05, Table 4). When evaluating the intake only from the six forage resources, the BG consumed less ADF, NDF, lignin, and all SC than the NG (*p* < 0.05, Table 4).

### 3.4. Associations between Selection, Intake, and the Nutritional Value of the Forage Species

In the NG, only the OM content had a positive association with DMI (r = 0.87, *p* = 0.010). Meanwhile, in the BG, the selection index and DMI exhibited a negative association only with the flavonoids content (r = −0.98 and r = −0.99, *p* = 0.001, respectively, Table 5).

Finally, for the DMI, significant relationships were only found between the CP and OM for NG, while for BG, the SI had a significant relationship with flavonoids and the DMI had a significant relationship with IVDMD and flavonoids (Table 6).

## 4. Discussion

The six evaluated plants had different biomass availability and SI in the tropical deciduous forest paddocks. The forages evaluated in the present study were chosen based on their different RIV, which is a measurement of their impact on the ecosystem. Hence, the novelty of this study was to compare simultaneously the use of plant resources that are not abundant in the field together with plant resources that are abundant in the field and are also consumed by goats (high DMI).

Under natural grazing conditions, multiple environmental factors affect the resource selection of goats. As a result, some plants could be underscored due to their low DMI or SI as a result of their low biomass availability in the grazing/browsing area. The DMI and SI results obtained from a cafeteria trial may allow evaluating the selection of goats without the confounding effects of biomass availability [28]. However, to our knowledge, such studies mainly evaluate plant/feed resources that are selected considering their high DMI or biomass availability.

### 4.1. Forage Selection of Juvenile Goats with Different Browsing Experience

In this study, the BG selected *P. piscipula* and *S. gaumeri* leaves (Table 2), which are highly available in the field as a resource and had an acceptable nutritional value [12,34], but also had lower flavonoid content (Table 1). Meanwhile, the NG did not display any selection pattern (Table 2). Similar to previous reports under grazing/browsing conditions, in the present cafeteria experiment, the BG had a high SI for *P. piscipula* and low SI for *G. floribundum*. In addition, *C. alliodora, C. gaumeri,* and *N. emarginata* had low SI (first report), while *S. gaumeri* had a high SI (first report). This highlights the importance of testing selection and intake without the confounding effect of biomass availability.

Ruminants can use two types of mechanisms to assess forages: (a) pre-ingestive mechanisms [44] and (b) post-ingestive mechanisms [45]. Sight, smell, and taste allow a sensory evaluation of resources. By including the learning factor, either through their peers or their mother [1], the goats gain experience and improve the use of available resources. The optimal outcome of experience can be seen as a balanced diet in terms of nutrients and SC to avoid any excesses or deficiencies as well as toxicity risks [34,46,47]. The latter leads to optimizing the nutrient harvest from their diet [48].

In the field, consumption of a diet with an excess of protein (N) implies an additional energetic cost to dispose of such excess [9]. Goats can also use another compensation mechanism to reduce the need to eliminate excess N in the urine by consuming plant species with high polyphenol content (such as TT and CT). The latter can bind with the N, and goats can void that excess N in the feces. Such behavior has also been reported in cafeteria trials [12,47]. However, in this study, the higher selection of *N. emarginata* foliage (34% CT) by the NG group, as compared to the BG, could not be explained as a mechanism to compensate for the excess of protein because both groups of animals do not differ in their CP intake (Table 4). The NG did not show any SI pattern and included in their diet all the evaluated forage resources equally. Hence, they even include those plants that could be classified as having “low nutritional value” due to their high SC contents, high fiber contents, low CP content, or even their low in vitro digestibility. Although SC, such as alkaloids, flavonoids, saponins, and phytoestrogens, among others, can also regulate intake and forage resource utilization by ruminants [49], it is likely that the lack of selection by NG in the present study was based on the “spread the risk” strategy [12]. According to the latter, toxin dilution or avoiding saturation of detoxification pathways could be important reasons why herbivores select a mixed diet [50].

Meanwhile, the BG selected *P. piscipula* and *S. gaumeri,* which had suitable nutrient content and lower SC content (CT and flavonoids). The latter could be due to their previous browsing experience. Thus, a trade-off between quality and quantity of available herbage may lead to herbivores selecting diets of intermediate quality in order to maximize their overall rate of nutrient assimilation [51] rather than avoiding secondary compounds.

The SI values found in the present work suggested that the forage intake and SI results from previous field studies with grazing goats [4,8] were likely influenced by the plant’s availability in the field. Meanwhile, in the present cafeteria experiment, differences found in the SI of the six resources between the NG and BG indicate that goats can better choose feeds when offered without restriction. However, the browsing experience played an important role. Thus, the NG selected all forages equally, while for the BG, the content of SC (flavonoids) had an influence on their SI.

The present results suggested that the forage availability may generate discrepancies in the SI results when forage resources are evaluated under field grazing/browsing conditions or even under controlled station situations such as cafeteria trials. This study demonstrated the interaction between animal experience, the chemical composition of plants (nutrients and SC), as well as biomass availability in the resulting forage SI. Thus, it allows us to better understand how animals balance their diets in relation to cost-benefit [48], reiterating that goats’ learning (experience) has more influence than the “innate” knowledge resulting from natural or artificial animal selection/breeding [21].

### 4.2. Forage Intake of Juvenile Goats with Different Browsing Experience

The intake results showed a similar trend to the SI results in the BG, suggesting that the integration of pre-and post-ingestive mechanisms, as well as previous experience, help animals to ensure the ingestion of diets with better quality [44,45]. Meanwhile, the NG consumed the six species in similar quantities (Table 3). As with SI, these results indicate that animals explored among the resources offered without a clear pattern towards the more nutritious resources. The latter was probably due to their lack of grazing/browsing experience and previous contact with the plant materials. The experience acquired as the animals grow allows them to vary their diet intending to better use the available resources [22] while aiming to meet their nutritional requirements [1]. The latter agrees with the present results when considering both the total intake from the whole diet and each forage alone. When considering only the forages, the BG consumed a lower quantity of fiber (NDF, ADF, and lignin) and SC (FT, TT, TC, alkaloids, flavonoids, and saponins) than the NG group (Table 4). When the whole diet was considered, only the total intake of SC was lower for the BG. Some factors that can help explain the results are: (i) the innate experience, (ii) the learning process achieved by observing their grazing counterparts, or (iii) the learning by self-experience while grazing/browsing [1,23,25].

The goats in both groups had a similar daily LWG throughout the study. This showed that, although both groups tried to meet their requirements, their intake and selection strategy varies for each resource, possibly due to experience.

### 4.3. Associations between Selection, Intake, and the Nutritional Value of the Forage Species

The intake and selection results in the BG showed that they searched for plants favoring those with the best nutritional quality while avoiding those plants that have high SC content. Flavonoids, resulted in a negative association with SI and DMI (r = −0.98 and r = −0.99, respectively, *p* = 0.001) in the BG, while the OM content was positively associated with DMI in the NG (r = 0.87, *p* = 0.010). The regression analysis for the BG showed that the best DMI predictors were flavonoids and IVDMD, while flavonoids were the best predictor for SI (Table 6). On the other hand, beneficial effects have been reported when consuming plants with low amounts of flavonoids in their diet as a supplement [13,14]. It is likely that pre-exposure of goats during grazing in the heterogeneous vegetation has created some level of awareness (knowledge) of flavonoids leading to differences in utilization [1].

Knowledge of how nutritional and behavioral factors interact could serve in the proposal of innovative management strategies aimed at modifying the behavior of herbivores to improve their own nutrition, health, and well-being, while also promoting the health and integrity of the ecosystems on which they depend.

In practice, the use of forages containing a diverse array of SC could be optimized by reducing the adverse effects of any of the compounds through the mixing of plants with adequate protein and energy levels (“spread the risk” strategy) in a similar fashion to plant selection and intake when animal browse freely in a biodiverse area. Feeding a diet that includes a variety of SC can allow the ruminal microorganisms to tolerate or adapt to the high total quantity of SC, as the risk of saturating specific detoxification pathways is reduced. In agreement, the intake of various forage species by goats has been reported, including species with low CP, low digestibility, and high amounts of CT, but the total diet harvested can satisfactorily meet the nutritional needs of ruminants [34,47].

The learning acquired through browsing/grazing helps ruminants to differentiate the resources they use, allowing them to discriminate between different nutritional values, including SC [1]. The lack of experience generates a similar use of resources, despite their nutritional value, resulting in a discrepancy between selection and intake. This was more evident in the NG, where no significant correlation was found for SI and DMI with plant chemical composition, but DMI was guided by macronutrient intake (CP and OM) (Table 6).

## 5. Conclusions

In the present study, it was demonstrated that goats with browsing experience guided their selection and intake towards forage resources of better nutritional quality when there was no restriction on their availability (cafeteria study). Goats with browsing experience also showed their ability to cope with secondary compounds (mainly flavonoids) and optimized their selection and intake of plants with high digestibility, such as *P. piscipula* and *S. gaumeri*. Meanwhile, the NG did not show a clear selection pattern.

The relevance of browsing experience implies that goats with prior exposure to different plant species containing different quantities of macronutrients and secondary compounds can better balance their diet by knowing what type of plant to select. This implies that some resources with low availability are not consumed due to the animals’ selection decision rather than the plant’s availability or its chemical composition. Naïve goats, on the contrary, may have more difficulties in harvesting plants that can result in an optimal balance between plant secondary metabolites and macronutrients.

Therefore, in the context of a heterogeneous environment that includes a large number of plant species with variation in availability and chemical composition, goats with browsing experience will be more capable to make more efficient use of resources and consequently optimize their productive performance.

## Figures and Tables

**Table 1:** Chemical composition ^a^, secondary metabolite content, in vitro dry matter and organic matter digestibilities, and the metabolizable energy of feeds offered to juvenile goats (Concentrate feed, *Pennisetum purpureum* grass, and the six experimental forage species).

	Feed (g/Kg DM)						
	Concentrate Feed	*P. purpureum* Grass	*Caesalpinia gaumeri*	*Cordia alliodora*	*Gymnopodium floribundum*	*Neomillspaughia* *emarginata*	*Piscidia* *piscipula*
DM	913.2	248.0	943.9	929.3	931.0	944.9	925.7
CP ^a^	165.2	74.1	108.1	143.2	133.1	153.5	144.8
EE ^a^	35.6	7.1	41.5	49.9	6.1	12.3	32.7
Ash ^a^	61.9	75.1	36.0	90.7	65.6	72.3	95.1
NDF ^a^	275.1	671.2	349.3	343.2	455.0	544.8	399.4
ADF ^a^	59.2	444.2	260.9	220.1	379.5	336.0	268.4
LIG ^a^	1.75	108.0	119.8	74.8	197.4	182.3	138.6
TP ^a^	0.7	2.1	41.5	29.5	74.5	61.9	24.6
TT ^a^	ND	1.4	23.3	16.2	60.6	42.8	6.9
CT ^a^	NE	ND	ND	ND	376.0	347.0	151.0
ALK ^a^	NE	NE	142.6	106.8	102.8	106.1	95.0
FLA ^a b^	NE	NE	49.9	42.8	47.8	49.4	17.4
SAP ^a c^	NE	NE	13.6	45.8	73.4	56.2	35.6
IVDMD ^a^	837.5	475.6	479.2	585.9	347.2	256.6	351.9
IVOMD ^a^	847.7	479.0	482.3	631.0	355.9	267.6	382.8
ME ^d^	12.7	7.0	7.4	9.1	5.3	3.9	5.5

^a^ g/kg DM except were stated; ^b^ equivalent to catechins; ^c^ equivalent to diosgenin; ^d^ ME (MJ/kg DM) estimate = 0.016 × digestible organic matter. DM: dry matter; CP: crude protein; EE: ether extract; NDF: neutral detergent fiber; ADF: acid detergent fiber; LIG: lignin; TP: total phenols; TT: total tannins; CT: condensed tannins, ALK: alkaloids; FLA: flavonoids; SAP: saponins; IVDMD: in vitro dry matter digestibility; IVOMD: in vitro organic matter digestibility; ME = metabolizable energy; NE: not estimated; ND: not detected.

**Table 2:** Effect of browsing experience of juvenile goats on the selection index (95% confidence intervals; 95% CI) of six forage resources evaluated in a cafeteria trial lasting six days of observations (30 min/day).

Foliage	Naïve Goats Group		Browser Goats Group		SEM
	Mean	95% CI	Mean	95% CI	
*Caesalpinia gaumeri*	0.139 ^A,a^	0.067–0.266	0.030 ^B,b^	0.009–0.092	0.0225
*Cordia alliodora*	0.090 ^A,a^	0.041–0.188	0.071 ^A,b^	0.031–0.156	0.0228
*Gymnopodium floribundum*	0.139 ^A,a^	0.066–0.270	0.064 ^A,b^	0.027–0.144	0.0267
*Neomillspaughia emarginata*	0.215 ^A,a^	0.108–0.382	0.049 ^B,b^	0.020–0.115	0.0297
*Piscidia piscipula*	0.083 ^B,a^	0.036–0.176	0.454 ^A,a^	0.274–0.647	0.0501
*Senegalia gaumeri*	0.182 ^A,a^	0.089–0.336	0.339 ^A,a^	0.186–0.534	0.0551
SEM	1.4561				

SEM; standard error of the mean. ^A,B^ Means with different capital letters between groups (rows) differ significantly (*p* < 0.05). ^a,b^ Means with different lowercase letters within each group (columns) differ significantly (*p* < 0.05).

**Table 3:** Effect of browsing experience of juvenile goats on dry matter intake (g DM/kg^0.75^) and 95% confidence intervals (95% CI) of the six forage resources evaluated in the cafeteria trial lasting six days of observations (30 min/day).

Foliage	Naïve Goats Group		Browser Goats Group		SEM
	Mean	95% CI	Mean	95% CI	
*Caesalpinia gaumeri*	3.350 ^A,a^	2.190–5.040	1.450 ^B,b^	1.160–2.210	0.0689
*Cordia alliodora*	2.300 ^A,a^	1.610–3.560	2.070 ^A,b^	1.490–3.200	0.0630
*Gymnopodium floribundum*	3.200 ^A,a^	2.080–4.910	1.830 ^A,b^	1.370–2.790	0.0674
*Neomillspaughia emarginata*	2.890 ^A,a^	1.860–4.640	1.750 ^A,b^	1.330–2.640	0.0675
*Piscidia piscipula*	2.260 ^B,a^	1.580–3.520	5.850 ^A,a^	3.770–7.740	0.0737
*Senegalia gaumeri*	3.250 ^A,a^	2.080–5.040	5.390 ^A,a^	3.510–7.260	0.0683
SEM	0.6597				

SEM: standard error of the mean. ^A,B^ Means with different capital letters between groups (rows) differ significantly (*p* < 0.05). ^a,b^ Means with different lowercase letters within each group (columns) differ significantly (*p* < 0.05).

**Table 4:** Effect of browsing experience of juvenile goats on the total intake (g/Kg^0.75^ ± standard deviation) of the whole diet (balanced feed + grass + forages) or the sum of the six forages resources (forage) evaluated in a cafeteria trial lasting six days of observations (30 min/day).

Item	Whole Diet		*p*-Value	Forage		*p*-Value
	Browser Goats Group	Naïve Goats Group		Browser Goats Group	Naïve Goats Group	
DM	72.920 (±13.440)	70.790 (±9.430)	0.439	16.192 (±4.962)	17.884 (±4.581)	0.137
CP	8.768 (±1.329)	8.561 (±0.988)	0.454	2.605 (±0.862)	2.698 (±0.754)	0.630
EE	1.568 (±0.219)	1.474 (±0.209)	0.067	0.552 (±0.186)	0.491 (±0.197)	0.178
OM	67.620 (±12.410)	65.860 (±8.740)	0.490	14.868 (±4.585)	16.652 (±4.293)	0.093
ADF	21.491 (±5.421)	20.738 (±3.809)	0.498	4.575 (±1.414)	5.449 (±1.375)	0.010
NDF	36.180 (±8.210)	34.876 (±5.779)	0.439	6.626 (±2.042)	7.815 (±1.910)	0.013
LIG	6.446 (±1.525)	6.500 (±1.158)	0.867	2.267 (±0.694)	2.717 (±0.693)	0.008
TP	0.582 (±0.168)	0.905 (±0.219)	0.001	0.493 (±0.160)	0.824 (±0.213)	0.001
TT	0.257 (±0.093)	0.557 (±0.164)	0.001	0.208 (±0.088)	0.513 (±0.160)	0.001
CT	1.791 (±0.701)	3.059 (±1.280)	0.001	1.791 (±0.701)	3.059 (±1.280)	0.001
ME ^a^	6.191 (±0.941)	5.991 (±0.644)	0.298	0.960 (±0.306)	1.042 (±0.313)	0.266
ALK	ND	ND		1.633 (±0.516)	1.957 (±0.513)	0.009
FLA	ND	ND		0.399 (±0.144)	0.698 (±0.176)	0.001
SAP	ND	ND		0.616 (±0.184)	0.791 (±0.183)	0.001

^a^ = in MJ/kg DM. CP: crude protein; EE: ether extract; OM: organic matter; NDF: neutral detergent fiber; ADF: acid detergent fiber; LIG: lignin; TP: total phenols; TT: total tannins; CT: condensed tannins; ME: metabolizable energy; ALK: alkaloids; FLA: flavonoids; SAP: saponins; NS: not significant; ND: not determinate.

**Table 5:** Correlation coefficients between chemical composition, secondary compounds content, in vitro dry matter and organic matter digestibility, metabolizable energy, foliage density of six forage resources and selection index (SI) and intake (DMI/Kg^0.75^) of juvenile goats with (BG) and without (NG) browsing experience during a cafeteria trial.

	SI				DMI/Kg^0.75^			
	Browser Goats Group		Naïve Goats Group		Browser Goats Group		Naïve Goats Group	
Item	C	*p*-Value	C	*p*-Value	C	*p*-Value	C	*p*-Value
CP	0.540	0.269	0.393	0.441	0.630	0.179	−0.043	0.936
EE	0.210	0.690	−0.474	0.342	0.239	0.648	−0.300	0.564
OM	0.523	0.286	0.423	0.403	0.502	0.309	0.878	0.021
ADF	−0.181	0.731	0.564	0.244	−0.181	0.731	0.514	0.297
NDF	−0.117	0.825	0.738	0.094	−0.112	0.832	0.192	0.716
Lignin	−0.039	0.941	0.584	0.223	−0.051	0.924	0.464	0.353
IVDMD	−0.214	0.684	−0.601	0.207	−0.195	0.710	−0.229	0.662
IVOMD	−0.164	0.755	−0.646	0.165	−0.150	0.776	−0.332	0.520
ME	−0.204	0.698	−0.618	0.191	−0.189	0.720	−0.260	0.619
FD	0.073	0.891	−0.125	0.812	0.149	0.777	0.368	0.473
TF	−0.641	0.170	0.462	0.356	−0.659	0.154	0.460	0.358
TT	−0.662	0.152	0.378	0.460	−0.681	0.136	0.408	0.422
CT	−0.205	0.696	0.380	0.457	−0.246	0.638	0.108	0.838
ALK	−0.552	0.256	0.100	0.850	−0.552	0.256	0.532	0.277
FLA	−0.987	0.001	0.287	0.581	−0.995	0.001	0.358	0.485
SAP	−0.252	0.629	0.163	0.758	−0.258	0.621	−0.091	0.864

C: coefficient; CP: crude protein; EE: ethereal extract; OM: organic matter; NDF: neutral detergent fiber; ADF: acid detergent fiber; IVDMD: in vitro dry matter digestibility; IVOMD: in vitro organic matter digestibility; ME: metabolizable energy; FD: foliage density; TP: total phenols; TT: total tannins; CT: condensed tannins; ALK: alkaloids; FLA: flavonoids; SAP: saponins.

**Table 6:** Relationship between chemical composition factors of forage resources with selection index (SI) and intake (DMI/kg^0.75^) in juvenile goats with and without browsing experience.

	Regression Equations	R^2^	R^2^_Ajusted_
NG ^a^			
DMI	=−21.04 (4.180) [0.024] + 0.0763 (0.033) CP [0.104] + 0.2456 (0.042) OM [0.010]	91.77	86.29
BG			
SI	= 0.6364 (0.039) [0.001] − 0.12186 (0.009) flavonoids [0.001]	97.51	96.89
DMI	= 8.806 (0.189) [0.001] − 0.01571 (0.003) IVDMD [0.027] − 1.3413 (0.030) flavonoids [0.001]	99.85	99.75

^a^ = No significant relationship was found for the selection index. NG: naïve goats group; BG: browser goats group; CP: crude protein; OM: organic matter; IVDMD: in vitro dry matter digestibility; values within brackets: standard error; values within square brackets: *p*-value for each factor. R^2^: R-square.

## Data Availability

Data supporting reported results will be provided upon request.
